# Reasons for using traditional and complementary care by people living with HIV on antiretroviral therapy and association with interrupted care: a mixed methods study in Eswatini

**DOI:** 10.1186/s12906-023-04184-5

**Published:** 2023-10-04

**Authors:** Marjan Molemans, Ria Reis, Fortunate Shabalala, Njabuliso Dlamini, Nelisiwe Masilela, Njabulo Simelane, Christopher Pell, Ariel Chao, Donna Spiegelman, Eva Vernooij, Frank van Leth

**Affiliations:** 1 Amsterdam Institute for Social Science Research, Department of Anthropology, University of Amsterdam, Amsterdam, The Netherlands; 2 Amsterdam Institute for Global Health and Development, Amsterdam, The Netherlands; 3 Amsterdam Public Health Research Institute, Amsterdam, The Netherlands; 4 Department of Public Health and Primary Care, Leiden University Medical Centre, Leiden, The Netherlands; 5 The Children’s Institute, School of Child and Adolescent Health, University of Cape Town, Cape Town, South Africa; 6 Department of Community Health Nursing, Faculty of Health Sciences, University of Eswatini, Mbabane, Eswatini Swaziland; 7 National Emergency Response Council on HIV and AIDS (NERCHA), Mbabane, Eswatini Swaziland; 8 Ministry of Foreign Affairs and Trade, Mbabane, Eswatini Swaziland; 9 Department of Global Health, Amsterdam UMC, location University of Amsterdam, Amsterdam, the Netherlands; 10 Yale School of Public Health, New Haven, USA; 11 Department of Interdisciplinary Social Sciences, Utrecht University, Utrecht, The Netherlands; 12 Department of Health Sciences, Vrije Universiteit, Amsterdam, The Netherlands

**Keywords:** Eswatini, HIV, Interrupted care, TCAM, Test-and-treat, Antiretroviral therapy, ART, HIV continuum of care.

## Abstract

The use of traditional, complementary, and alternative medicine (TCAM) can lead to delays and interruptions in the HIV continuum of care. This study explores reasons for TCAM use in people living with HIV on antiretroviral therapy (ART) in Eswatini and compares interrupted care between different types of TCAM users. Data were collected using surveys in the MaxART study (a test-and-treat trial) between 2014 and 2017 to assess the exposure, namely visiting a TCAM provider. Additionally, visit dates were retrieved from clinic records to assess the outcome, interrupted care. Open-ended questions were analysed with qualitative content analysis (n = 602) and closed questions with bivariable and multivariable analysis (n = 202). Out of 202 participants, 145 (72%) never used TCAM, 40 (20%) ever used, and 17 (8%) is currently using TCAM (diviners, herbalists, and religious healers). No differences in interrupted care were found comparing never (reference category), past (Odds Ratio: 1.31, 95% confidence interval: 0.63–2.72), and current users (1.34, 0.47–3.77), while adjusting for gender, time since HIV diagnosis, and time on ART. Contextual factors affecting the choice for TCAM were the influence of family, advice from the health facility, and religious beliefs. Individual factors include trust in biomedical care, type of illness, no need for additional care, and practical reasons such as financial means. In conclusion, individual and contextual factors influence the choice for TCAM. Interrupted care does not differ between never, past, and current users.

## Background

There is mixed evidence on the effect of medical pluralism on the HIV continuum of care. Use of traditional, complementary, and alternative medicine (TCAM) can be a barrier in this continuum [1], as it can lead to delayed HIV diagnosis and/or treatment initiation [2–5] and interruptions in care [6]. Treatment interruptions can lead to clinical disease progression [7, 8] and antiretroviral drug resistance [9]. A study in Zambia described how some patients reported discontinuing antiretroviral therapy (ART) because of TCAM use [10]. The use of herbal remedies was associated with nonadherence to ART in South Africa, while the use of TCAM decreased after ART initiation [11]. However, traditional healers also refer patients to biomedical healthcare and take precautions to avoid drug interactions with ART [12]. Other studies have found no association between TCAM use and ART initiation and between the use of herbal medicine and ART adherence [13, 14].

Most research was conducted before the global implementation of the WHO’s recommendation of immediate ART initiation at HIV diagnosis (“test-and-treat”) [15], including a review reporting mixed evidence on the association between ART adherence and TCAM. This review did not include studies from sub-Saharan Africa. Research alongside a “test-and-treat” trial in KwaZulu-Natal, South Africa, showed that people living with HIV (PLWHIV) continue to seek help concurrently with the biomedical and traditional healing systems, even when there is broad availability of ART [16].

Reasons for TCAM use are diverse. Sociocultural, religious, and spiritual values regarding health and disease play a role [17]. Participants in a “test-and-treat trial” in KwaZulu-Natal used the two systems for different needs, where traditional healers were consulted for types of afflictions that could not be addressed by the biomedical system [16]. This idea that the biomedical system and TCAM cater to different needs is also clear in three other studies where participants similarly reported using TCAM for specific illnesses, mostly with a spiritual cause [16, 18, 19]. The patients’ social network is also cited [17], with an important role for family expectations [16]. Specifically in Eswatini, some PLWHIV reported pressure from family or community members to use non-biomedical treatments instead of ART [20]. TCAM is also used for health improvement, e.g., boosting immunity, increasing appetite, and decreasing fatigue [21]. Boosting CD4 count and healing HIV were also reported in South Africa, although most participants explained that they used TCAM for reasons not related to their HIV status [18]. In some aspects, TCAM is perceived as better care: more privacy [19], care and support, autonomy over one’s health, and trust [17]. Lastly, practical advantages are mentioned, such as low cost and better accessibility [17].

We conducted a mixed methods study to explore reasons for TCAM use in people living with HIV on antiretroviral therapy and to compare the frequency of interrupted HIV care between users and non-users of TCAM. Our study took place alongside a “test-and-treat” implementation study, MaxART, in Eswatini. In 2019, Eswatini reported an HIV prevalence of 27% among adults aged 15–49 [22]. The country’s healthcare system is pluralistic and consists of a biomedical sector and TCAM providers. The biomedical sector is headed by the Minister of Health at the national level [23]. TCAM providers are composed of two types, those practicing traditional medicine (herbalists and diviners) and religious healers from various Evangelical and African healing churches [24]. Despite the existence of a Traditional Healers Association since the 1980s, traditional healers are not formally embedded in a professional structure under or besides the Ministry of Health. Their status as practitioners of ancestral knowledge earns them support from traditional leaders. However, this status also leads to contestation, for instance, from Evangelical (born again) and mainstream Christian churches. This contestation also extends to African healing churches as they incorporate traditional beliefs [24].

## Methods

### Setting

This study draws on data from the social science and the clinical sub-studies of the multidisciplinary MaxART study, which assessed the impact of the “test-and-treat” strategy in Hhohho region, Eswatini. The study’s protocol and the social science sub-study are described in detail elsewhere [25, 26]. Briefly, the MaxART study implemented a “test-and-treat” intervention for all HIV-positive, ART-naive individuals, excluding pregnant and breastfeeding women, attending one of the 14 participating facilities. The MaxART study used a stepped-wedge design, where facilities transitioned from control (national ART guidelines with CD4 threshold) to intervention (test-and-treat). The social science sub-study was conducted in nine of 14 facilities, purposefully selected as a diversity sample on patient volume and setting and based on previous research in these facilities [27].

### Participants

**Fig. 1:**
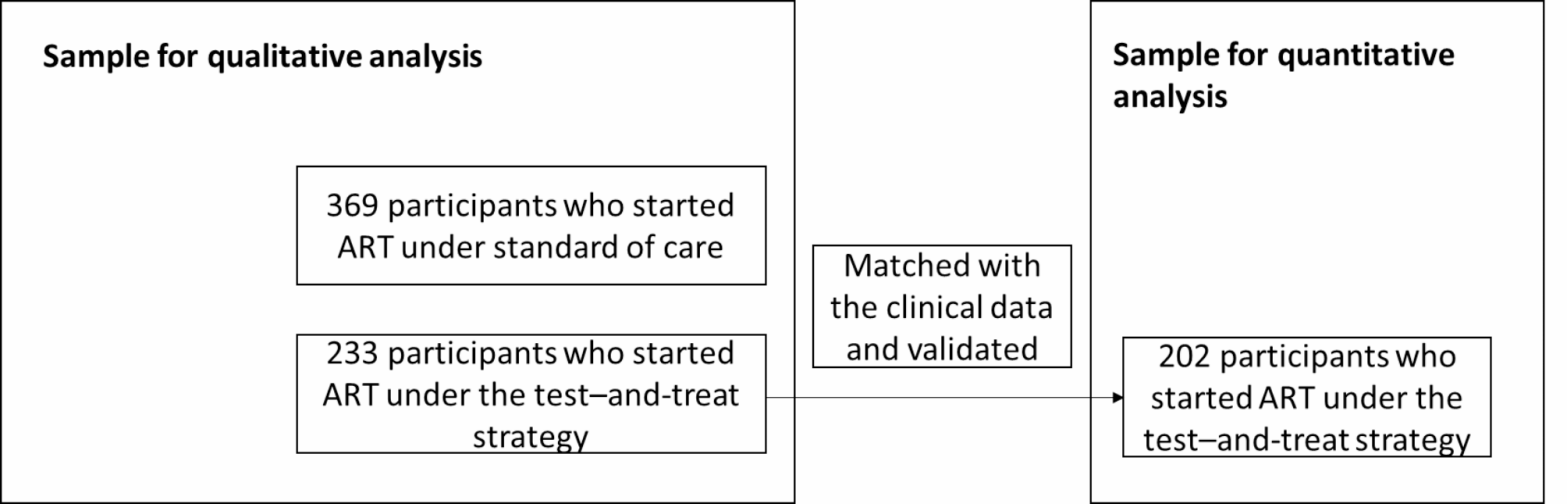
Flow diagram of the different samples in the study

This study used data from two partly overlapping samples of participants (Fig. 1). The sample for quantitative analysis, which looks into the association between TCAM use and interrupted care, consists of a matched sample between the participants of the social science sub-study and the clinical sub-study. This resulted in 219 matched participants, of which 17 could not be validated, resulting in 202 participants. These 202 participants were interviewed after they initiated ART under the test-and-treat intervention.

Information on reasons for TCAM use was available for all participants in the social science sub-study. The social science sub-study had one round of interviews before and one round after the introduction of test-and-treat. Therefore, the sample for qualitative analysis consists of 369 participants who were interviewed after they initiated ART under the national guidelines on ART eligibility (CD4 count above 350 cells/mm^3^) and 233 who were interviewed after they initiated ART under the test-and-treat intervention.

### Sample size considerations

With a sample size of 202 (for the quantitative analysis), we can calculate a minimal detectable effect assuming 80% power and an alpha of 0.05. Non-retention in the MaxART study was 20% in the group following national guidelines and 14% in the “test-and-treat” group [28]. We assumed that interrupted care would be more frequent than non-retention. With the observed exposure distribution, we were able to detect an odds ratio of around 2.5 as being statistically significant.

### Data collection

The social science sub-study used a questionnaire with open- and closed-ended questions. The survey was conducted by trained research assistants during face-to-face interviews in Siswati. The answers in Siswati were first collected on paper and then entered into MS Excel® and translated by the research assistant who conducted the interview. Data entry and translation were cross-checked, and inconsistencies were discussed [26].

From the clinical sub-study, we only used scheduled and actual visit dates. These were extracted from routine clinical records and transferred to an electronic database. Data management and quality assessment are described elsewhere [28].

Data collection for the sample for the qualitative analysis took place from September 2014 to June 2017 and for the sample for the quantitative analysis from April 2015 to August 2017.

### Data analysis

Answers to open questions were analysed with qualitative content analysis to explore reasons for TCAM use. Answers to closed questions were analysed using bivariable and multivariable regression analysis to assess the association between use of TCAM and interrupted care. The exposure, use of TCAM, was assessed by two questions: “Before you went to the facility where you tested HIV positive most recently, have you visited any of the following service providers for help?” and “Since you have started ART, have you visited any of the following service providers for help?”. Both questions had the following answer options: diviner, herbalist, religious healer, pharmacy, self-medication, none, and other. Both were followed by “why?” to assess the reasons for use. The outcome of interrupted care was defined as being more than ten days late to an appointment or having the last visit 120 days before the study end or date of death, whichever came first. Missing data were not imputed. We performed a bivariable logistic regression with groups of TCAM users and interrupted care. In the multivariable analysis, we adjusted for gender, time since HIV-positive diagnosis, and time on ART. Additionally, we performed an exploratory factor analysis on ever visiting a diviner, herbalist, and religious healer with the patients included in the quantitative analysis to explore a possible underlying latent concept that can be explained by these variables. For the qualitative analysis, we used a deductive coding approach. The quantitative analysis preceded the qualitative analysis, but as data collection occurred at the same time, the quantitative analysis did not inform the qualitative analysis. Quantitative analysis was performed using Stata® 16.1, and qualitative analysis was conducted with ATLAS.ti® 9.

### Ethical review

The Swaziland National Health Research Review Board approved the MaxART study, including the social science sub-study, in July 2014 (MH/599 C/FWA00015267). Participants provided informed consent for the MaxART study and additionally written consent for the social science sub-study.

## Results

### Background characteristics of the study sample

The characteristics of the participants included in the quantitative analysis are described in Table 1. The majority of the participants were female (78%). The median age was 35 years. Most received only primary or secondary education. The main religion was African Healing Churches (54%). The sample characteristics for the qualitative analysis are very similar (Table 2).

**Table 1:** Background characteristics of the sample for quantitative analysis, n = 202

	n (%)
Age, median (IQR)	35 (29–45)
Gender	
Male	45 (22)
Education	
No formal education Primary Secondary Higher Other	40 (20) 60 (31) 67 (33) 31 (15) 2 (1)
Religion	
African Healing Churches Evangelical Mainstream Christian None Other	109 (54) 63 (31) 11 (5) 18 (9) 1 (1)
Marital status	
Currently in a relationship Divorced/ widowed/ separated Single / never married	162 (81) 19 (9) 20 (10)
Time since HIV positive in years	
<=1 1–5 > 5	68 (34) 79 (39) 54 (27)
Time on ART in months	
<=3 3–6 > 6	35 (18) 61 (31) 101 (51)
Travel time to facility in minutes, median (IQR)	45 (30–60)

**Table 2:** Background characteristics of the sample for qualitative analysis, n = 602

	n (%)
Age, median (IQR)	35 (28–43)
Gender	
Male	138 (23)
Education	
No formal education Primary Secondary Higher Other	100 (17) 209 (35) 175 (29) 108 (18) 10 (2)
Religion	
African Healing Churches Evangelical Mainstream Christian None Other	321 (53) 184 (31) 33 (5) 57 (9) 7 (1)
Marital status	
Currently in a relationship Divorced/ widowed/ separated Single / never married	519 (86) 50 (8) 32 (5)
Time since HIV positive in years	
<=1 1–5 > 5	278 (47) 198 (33) 121 (20)
Time on ART in months	
<=3 3–6 > 6	175 (29) 178 (30) 242 (41)
Travel time to facility in minutes, median (IQR)	40 (30–60)

### Use of TCAM

Participants in the quantitative analysis (n = 202) were divided into three groups never (72%), past (20%), and current (8%) TCAM users. Participants reported ever visiting the following TCAM providers: diviners (9%), herbalists (10%), and religious healers (11%). Some only used one type, and others visited different types of TCAM providers.

The sample for the qualitative (n = 602) consists of 73% never, 18% past, and 9% current users. Of these participants, 10% ever visited a diviner, 7% an herbalist, and 15% a religious healer.

### Quantitative results

#### Association between the use of TCAM and interrupted care

Of the 202 participants in this sample, 101 (50%) met the study’s definition of interrupted care.

No differences in interrupted care were found comparing never, past, and current TCAM users in either the bivariable or multivariable analysis controlling for sex, time since HIV positive, and time on ART (Table 3).

**Table 3:** Association between types of TCAM users and interrupted care, n = 202

	Interrupted care			
	Odds ratio (95% CI)	p-value	Adjusted odds ratio (95% CI)	p-value
Type of TCAM user				
Never user	Ref.		Ref.	
Past user	1.31 (0.65–2.64)	0.452	1.31 (0.63–2.72)	0.465
Current user	1.21 (0.44–3.30)	0.716	1.34 (0.47–3.77)	0.577
Gender, female			0.85 (0.41–1.75)	0.663
Time since HIV-positive diagnosis, in years				
<=1			Ref.	
1–5			0.70 (0.35–1.40)	0.316
>5			0.91 (0.42–1.97)	0.808
Time on ART, in months				
<=3			Ref.	
3–6			0.56 (0.24–1.33)	0.188
>6			0.57 (0.41–1.75)	0.184

The exploratory factor analysis with the variables ever use of diviner, herbalist, and religious healer did not indicate an underlying latent concept (uniqueness, which refers to the degree of variance in a variable that is specific to that variable alone and not shared with other variables, ranged from 0.91 to 0.94).

### Qualitative results

#### Reasons for use and non-use of TCAM

Reasons for TCAM use were categorized into individual and contextual factors. However, one’s individual factors are also influenced by contextual factors.

Contextual factors include the influence of family, health facility, and religion. Individual factors include the type of illness, no need for additional care, practical reasons, and trust in biomedical care.

##### Family

Both for use and non-use of TCAM, participants often indicated that they follow the preferences of the family in which they were raised. Some female participants also reported that their husbands played a role in whether to use TCAM.

*“I was raised in a place where they do not use it” (Male, never user, 38)*.

*“My husband has refused for me to take timbita [traditional medicine: herbal concoction] because I am taking treatment” (female, never user, 35)*.

##### Health facility

A frequently reported reason for not using TCAM was that ART should not be combined (“mixed”) with traditional medicine. Some respondents explicitly stated that they were told at the health facility to refrain from traditional medicine or visit TCAM providers, whereas others did not mention where they got the information.

##### Religion

Religion is a theme that emerged frequently, as it is cited as a reason both for and against TCAM use.

*“My religion doesn’t allow me” (female, never user, Evangelical, 40)*.

*“I was keeping church orders by taking spiritual medicines” (female, current user, African Healing Churches, 28)*.

These quotes reflect different stances between types of churches, with some African healing churches providing herbal medicine and holy water (water blessed by the priest) to be taken alongside ART.

Some participants made a clear distinction between traditional healers and religious healers.

*“I do not believe in traditional healing, but I do visit religious healers” (female, current user, 35)*.

*“From traditional healers, I am avoiding the traditional medicines whereas religious leaders do not give you anything to take” (male, current user, 23)*.

##### Type of illness

To a large extent, participants differentiated between illnesses requiring biomedical care and illnesses requiring TCAM. Some report seeking help from TCAM providers only for illnesses not related to their HIV status. The distinction is less clear for others, as people also report consulting them for headache, backache, or the feeling of losing strength.

*“I decide where to go to depending on the sickness I have which service provider to visit” (male, current user, 40)*.

*“I use traditional medicine for some sicknesses not related to HIV” (female, current user, 30)*.

That there is a distinction between types of illnesses requiring TCAM or biomedical care is also clear from statements that TCAM cannot help with certain illnesses.

*“I was not sick with something that needed traditional healers” (female, never user, 32)*.

Witchcraft-related illnesses and illnesses that the participant did not understand were seen as a type of illnesses that required TCAM.

*“I was strangled by something at night in my sleep” (female, current user, 44)*.

*“Pills do not aid with witchcraft-related illnesses” (female, current user, 67)*.

*“Because I did not know what I was sick with” (female, past user, 57)*.

A few participants indicated pregnancy as a condition requiring TCAM.

*“Because they said while I was still pregnant that I must use traditional method” (female, past user, 54)*.

What pertains to “sickness” differed between biomedicine and traditional healing, the latter also addressing misfortune in a broader sense. TCAM was also used for accomplishing business endeavours and addressing bad luck, ancestral spirits, and personal and social problems.

##### No need for additional care

Some respondents indicated additional care was not needed, as they were healthy, or the pills helped them.

Participants who used TCAM in the past and are no longer using it explained that they are “educated now”, feel better since starting treatment, or stopped using TCAM because of their HIV diagnosis.

##### Practical reasons

Some respondents reported practical barriers, such as not having financial means. This was only mentioned by respondents who are not using TCAM.

##### Trust

Some people indicate that they trust biomedical care more or that they distrust TCAM.

## Discussion

Drawing on data collected alongside the MaxART study in Hhohho, Eswatini, this study showed a low frequency of TCAM use in PLWHIV on ART in this setting. Those who use TCAM consult diviners, herbalists, and religious healers for varying reasons. We found no evidence of an association between TCAM use and interrupted HIV care. Reasons for TCAM use are diverse. As contextual factors, we identified influence from the family, the health facility, and religion. Individual factors included “no need for additional care”, trust, practical reasons, and “type of illness”.

Few studies quantified the proportion of PLWHIV on ART using TCAM. A systematic review of TCAM use in Sub-Saharan Africa reported a mean of 45% (17.6–62) of PLWHIV visiting a TCAM provider before the global implementation of the “test-and-treat” strategy [17]. Our finding of 27% ever or currently using TCAM is thus lower. This might be due to our definition of TCAM relying on visiting a provider, whereas others also include self-administered herbal medicine.

Research in neighbouring South Africa showed that consulting a traditional healer in the past year was associated with worst treatment outcomes [29]. The difference can lie in the different outcome, which was a missed or delayed visit in our study, while it discontinued treatment or a viral load higher than 400 copies/ml. Secondly, the timeframe, which was six months in South Africa, while in our quantitative sample, only half of the participants had been on ART for six months or longer.

The contextual factors align with the influence of the social network, which was identified in a systematic review of TCAM use [17]. Family and religion were also reasons for consulting TCAM in people on ART in Kenya [30]. Religion seems to play a complex role in our study setting, as it is a reason both for choosing TCAM and for not choosing it.

The individual factors are not completely in line with the literature. The factor “types of illness” is in line with Appelbaum Belisle et al. [18], who showed that patients use traditional African medicine and ART for different needs. However, our study shows a grey area, where participants report general discomforts that could be linked to their HIV status as a reason to make use of TCAM. Where other studies found that patients chose to use TCAM because they trust TCAM providers more and because the cost is lower [17], we found these two reasons only as explanations for not using TCAM. This might be because this is very context-specific, and the systematic review did not include studies from Eswatini. In addition, the ART provided in Eswatini is free of charge, which might have contributed to people not using TCAM because of the cost.

The message that “ART and traditional medicine should not be mixed”, was also often cited in our study, but a study in Kenya showed that this often ends up being counterproductive for the sustained use of ART, as patients subsequently interrupt ART to take traditional medicine [30]. Research in Eswatini and South Africa shows that patients feel reluctant to disclose TCAM use to biomedical healthcare providers [11, 31].

Therefore, we recommend healthcare workers not to tell PLWHIV on ART to refrain from using TCAM but to nuance this message so that it can be used for needs not related to their HIV status, with the highlight that general discomforts can be related to their HIV-status. Engaging in an open and informed dialogue would encourage patients to share their doubts and discomforts for which they make use of TCAM. It may offer opportunities to discuss what kind of traditional treatments interact negatively with ART (e.g., enemas, or specific drug-drug interactions), but also which treatments are harmless or beneficial, for instance, by supporting patients to come to terms with their diagnosis and persevere in their HIV-care.

The strengths of this study are that the mixed methods approach allowed us to look at different aspects of TCAM use among PLWHIV on ART in Eswatini during the implementation of the “test-and-treat” strategy. The link between the social science study and the clinical study gave us information on interrupted care. However, there are also some limitations. Firstly, the survey did not allow for much probing. For example, some participants just replied that they do not use TCAM, whereas further probing would have been of added benefit. Secondly, there is a risk of socially desirable answers, considering the interviews took place in a biomedical care setting. This would have led to an underestimation of the true prevalence of TCAM use in this setting. Third, we could only follow engagement in care for a limited period, meaning that some of the respondents had not yet had the opportunity to make use of TCAM because they had not been sick. Therefore, it is likely that the prevalence of TCAM use would be higher with a longer follow-up period. The fourth limitation is the definition of interrupted care, defined as being late to a visit. However, it is possible that participants borrowed or got medication elsewhere. Another limitation is the definition of TCAM, specifically regarding the inclusion of religious healers in this category. As shown in the analysis, there is a clear distinction for some respondents, while not for others. However, we chose the follow the participants’ wording in this. In Eswatini, the Evangelical and mainstream Christian churches play a role in this, as they place the African Healing churches under TCAM due to the belief in ancestors and witchcraft. In future studies, it would give more insight to ask participants what they understood under TCAM and what the role of religious healers in this is.

## Conclusions

The majority of PLHIV included in MaxART, a “test-and-treat” implementation study in Eswatini, does not use TCAM. Individual and contextual factors influence the choice for or against TCAM. No difference in interrupted care was found between current, past, and never users.

## Data Availability

All data analysed during this study are included in this published article.
